# Replacement of traditional prothrombin time monitoring with the new Fiix prothrombin time increases the efficacy of warfarin without increasing bleeding. A review article

**DOI:** 10.1186/s12959-021-00327-1

**Published:** 2021-10-15

**Authors:** Pall T. Onundarson, Ragnar Palsson, Daniel M. Witt, Brynja R. Gudmundsdottir

**Affiliations:** 1 Central Laboratory/Hematology, Landspitali National University Hospital of Iceland and University of Iceland Faculty of Medicine, Hringbraut, 101 Reykjavik, Reykjavik, Iceland; 2 Nephrology Division, Department of Medicine, Massachusetts General Hospital, Boston, MA USA; 3 Department of Pharmacotherapy, University of Utah College of Pharmacy, Salt Lake City, UT USA; 4 Central Laboratory/Hematology, Landspitali National University Hospital of Iceland , Reykjavik, Iceland

**Keywords:** Warfarin, Oral anticoagulation, Monitoring, Fiix, Prothrombin time

## Abstract

The antithrombotic effect of vitamin K antagonists (VKA) depends on controlled lowering of the activity of factors (F) II and X whereas reductions in FVII and FIX play little role. PT-INR based monitoring, however, is highly influenced by FVII, which has the shortest half-life of vitamin K-dependent coagulation factors. Hence, variability in the anticoagulant effect of VKA may be partly secondary to an inherent flaw of the traditional monitoring test itself. The Fiix prothrombin time (Fiix-PT) is a novel test that is only sensitive to reductions in FII and FX and is intended to stabilize the VKA effect. Two clinical studies have now demonstrated that when warfarin is monitored with the Fiix-PT based normalized ratio (Fiix-NR) instead of PT-INR, anticoagulation is stabilized and less testing and fewer dose adjustments are needed. Furthermore, the relative risk of thromboembolism was reduced by 50–56% in these studies without an increase in major bleeding.

## Introduction

From their advent, 70 years ago, the effect of vitamin K antagonists (VKAs) has been monitored by measuring the prothrombin time (PT), either Quick or Owren type [1, 2], that equally reflect reductions in vitamin K-dependent (VKD) coagulation factors (F) II, VII or X but not FIX [3, 4] to effectively prevent and treat thromboembolism (TE) [5, 6]. However, the pharmacodynamic effect of PT-monitored warfarin (hereafter referred to as PT-warfarin) is quite variable in many patients. International standardization of PT ratios for the purpose of VKA monitoring, leading to the international normalized ratio (INR, hereafter referred to as PT-INR) [7], has not reduced intra-individual anticoagulation variability that is mainly blamed on food and drug interactions and patient non-adherence.

In this article, we argue that the reasons for using the PT to monitor VKAs are primarily historical. Based on knowledge that accumulated after the development of the PT, it can be questioned whether the most relevant anticoagulant effect of VKAs has been monitored throughout the decades of their use. This article reviews recent data suggesting that the PT-INR suboptimally reflects the anticoagulant effect of warfarin and that anticoagulation outcomes can be improved considerably by monitoring a different effect, namely only the influence of FII and FX [3, 8, 9] while ignoring FVII and FIX.

## Efficacy and safety of current oral anticoagulants

In accordance with recent clinical guidelines [10, 11], the unmonitored newer direct oral anticoagulants (DOACs) have increasingly replaced warfarin as first-line anticoagulants in non-valvular atrial fibrillation (AF) and venous thromboembolism (VTE). In addition to convenience, this practice-shift is mainly based on pharmaceutical industry initiated clinical trials that have concluded that DOACs are at least similarly effective as warfarin that is dosed based on traditional PT-warfarin monitoring and that DOACs carry lower risk of intracranial hemorrhage [12, 13]. However, PT-warfarin has been found to be more effective and safer than DOACs in high thrombogenic-risk patients with mechanical heart valves [14] or triple-positive antiphospholipid antibody syndrome [15–17] and possibly as well following anterior wall myocardial infarction [18]. If PT-warfarin has advantages over DOACs in high-risk patients, why not also in patients that are at lower risk? Poor warfarin management, i.e. low time within target INR range (TTR), associates with high risk of TE, bleeding and mortality [5, 19–21]. Is it possible that poor PT-warfarin management or other quality issues in the control groups of the DOAC trials (e.g. low TTR) influenced the study outcomes? Some published data suggest so [22–24]. Furthermore, two real-world practice studies involving 130,911 and 196,061 patients have suggested that PT-warfarin may actually be more effective in clinical practice than are the DOACs. The authors of those studies suggest that the benefit of reduced intracranial bleeding with DOACs has been overemphasized as it may be at the cost of more thromboembolism due to lower anticoagulation level [25, 26].

There is little argument that DOACs have the major advantage of not needing to be routinely monitored. However, warfarin patients with very stable INR control can safely go up to 12-weeks between PT-INR tests [27]. Therefore, if the effectiveness and stability of warfarin could be further improved then further comparative effectiveness studies would at least seem warranted.

### Prothrombin time based VKA monitoring; history

The Quick-PT, invented in 1935 [1], in its original form mixed undiluted thromboplastin of rabbit brain origin and calcium chloride into citrated patient plasma and the ensuing clotting (“prothrombin”) time was measured. At the time, it was only known to be affected by a reduction in either of two then known coagulation factors, i.e., fibrinogen (FI) or prothrombin (FII), only the latter being a VKD factor. PT monitoring made VKA use possible in humans around 1950. Two new factors influencing the PT had then just been discovered, the non-VKD FV [28] and proconvertin (FVII) [2, 29]. For the purpose of VKA monitoring, Owren modified the PT in order to eliminate the influence of reduced non-VKD factors (fibrinogen or FV) on the PT [2]. Two further VKD coagulation factors, FIX and FX, only the latter of which influences Quick PT and Owren PT, were described in the 1950s [30, 31]. Over the ensuing decades the common wisdom was that an equal reduction in all VKD factors was necessary for full VKA anticoagulation, although it was considered sufficient to monitor only reductions in three out of the four, namely FII, FVII and FX.

Thromboplastins from different sources have different “strengths”; a strong thromboplastin has a short clotting time and does not detect the VKA effect as accurately as the more “sensitive thromboplastins” that have longer clotting times. Due to major differences observed in PT ratios, obtained from the same test plasmas from patients managed with VKAs with different thromboplastins, PT-ratios were later standardized as international normalized ratios (INR, PT-INR) [7]. This finally made the anticoagulation level comparable between clinical laboratories despite use of different thromboplastins and instruments [7]. Nevertheless, the use of a sensitive thromboplastin is generally recommended for VKA monitoring. The implementation of PT-INR worldwide improved the efficacy, safety and comparability of VKA management but did not solve the problem of high intra-individual variability.

### Target ranges and the therapeutic window

VKAs need monitoring of their anticoagulant effect. Although inconvenient, monitoring has benefits such as improving drug adherence [32] and providing the ability to identify food and drug interactions that remain obscure for the DOACs. Measuring a biological effect in blood has also led to an understanding of the relationship between PT-INR variability, TE and bleeding and it is universally accepted that VKAs have a narrow therapeutic window [5]. Over the decades, a consensus has been reached empirically on standard (2.0–3.0) and high intensity (2.5–3.5) PT-INR target ranges, the latter being used mainly for those with mechanical heart valves [5]. A low and in the authors’ opinion too narrow target range of 1.5–2.0 was shown to be less effective than a range of 2.0–3.0 in VTE [33]. Although a target range is a different concept from a therapeutic range, calculating the time that the PT-INR stays within specified target ranges (TTR) has turned out to be a useful surrogate measure of likely efficacy and safety during VKA therapy [19, 21]. Other informative variability measures include variance growth rate (VGR) [34–36], monitoring testing frequency, and dose change frequency [36, 37].

### VKA dose

The daily dose of warfarin varies from 0.5 to 20 mg daily between patients and this causes problems during initiation [38]. The dose is influenced by hereditary metabolic differences in sensitivity to VKA action but the daily dose requirement is also influenced by vitamin K supply from food and colonic bacteria [5, 6, 38]. However, the likely dose range needed for each patient becomes evident during the first treatment weeks. Once the dose range is established the main difficulty becomes variable PT-INR that influences the TTR.

Factors adversely influencing the ability to maintain stable warfarin dosing include geographical and cultural differences (e.g. distance from clinical laboratories, health insurance issues, dietary practices) and patient factors such as drug interactions, genetic factors, female gender, age, presence of chronic disorders such as heart failure, variable vitamin K intake, high PT-INR target (≥3), sudden change in lifestyle and poor adherence. Factors that associate with high TTR and favorable clinical outcome include dosing by specialized staff, self-management using portable INR monitors, formal dosing algorithms, and use of dosing software [5, 6]. Finally, studies on the value of genotyping cytochrome P450 2C9 or the VKOR genes at the initiation of VKA therapy have yielded conflicting results and continue to be debated [38, 39]. Although predictive of dose, the usefulness of genotyping during long-term anticoagulation is unknown.

### PT-INR variability

The variable anticoagulant effect of VKAs as indicated by the PT-INR may be the single most problematic feature of VKA management following the initiation phase. PT-INR variability is assumed to correctly reflect antithrombotic effect variability and, therefore, leads to dose adjustments, repeat testing and further dose adjustments. Furthermore, variable PT-INR associates with occurrence of both TE and bleeding [34, 35, 37]. PT-INR variability is usually blamed on food and drug interactions or patient non-adherence [5, 6, 40]. The question has, however, been raised whether the perceived variability in the antithrombic effect when measured as PT-INR could be partly due to the PT-test being affected by a factor that confounds anticoagulation assessment and dosing, namely FVII.

**Fig. 1:**
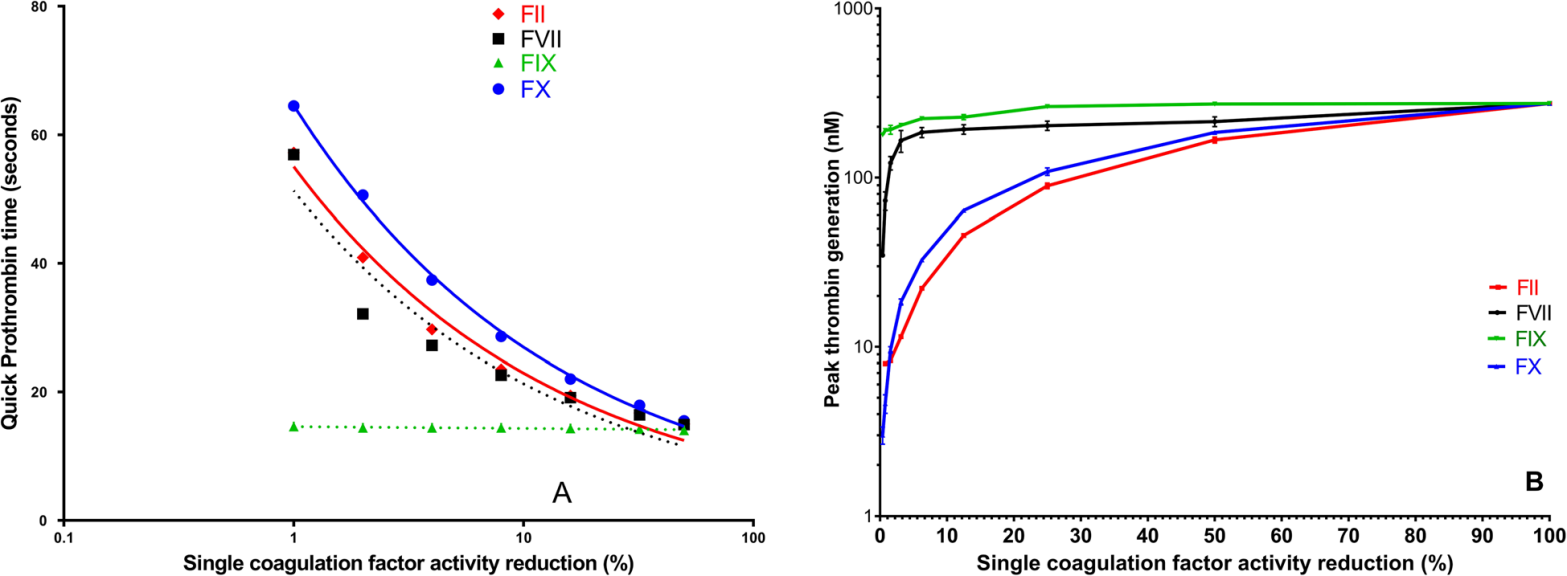
Influence of selective reduction in vitamin K dependent factors, a single factor at a time while others remain within normal range, on the undiluted Quick prothrombin time (Neoplastin with ISI 1.3, Stago, Asnieres, France: panel A) and and automated peak thrombin generation with clotting activated with a diluted thromboplastin (Neoplastin diluted 1:17,000: panel B) The figures are reproduced with permission from Thrombosis Research 2012;130:674–81 [3] and Journal of Thrombosis and Haemostasis 2016;15:131–9, respectively [41].

The PT-INR is similarly affected by reduced concentrations of FII, FVII or FX (Fig. 1A) [3]. The very short half-life of FVII (4–6 h) leads to this particular factor having a major influence on the PT-INR in the short term, e.g. during initiation, after dose changes, and when dose changes are made repeatedly within short time intervals. As further discussed later, it should be noted that during stable PT-warfarin management the percent normal activity levels of FII, FVII, FIX and FX differ considerably, i.e. FII 27 (95% range 15–40), FVII 48 (18–79), FIX 61 (32–89) and FX 15 [11–19] [41]. The activity range variation is higher for FVII and FIX which may reflect their shorter half-lives, 4–6 h and 21–30 h, respectively, than those of factors and II and X, 42–72 h and 27–48 h, respectively.

### The antithrombotic effect of warfarin

The assumed need to reduce all VKD coagulation factors similarly to obtain a full anticoagulant effect of VKAs has been questioned for decades. First, in the 1980’s Furie and Furie published studies suggesting that monitoring the native prothrombin antigen was a more effective monitoring method of warfarin than the PT ratio which was, however, poorly standardized at the time [42, 43]. Second, in vitro experiments demonstrated that thrombin generation was linearly dependent on the prothrombin concentration at any activity level and also dependent on FX concentration at activity levels < 25–30% but almost independent of the activity of FVII and FIX unless their activity was markedly reduced to << 5% [8]. Third, animal experiments demonstrated that the antithrombotic effect of warfarin depends on reductions in FII and FX and that reductions in FVII and FIX have little role at concentrations expected during therapeutic VKA anticoagulation [9]. Finally, these conclusions were confirmed by in vitro experiments suggesting a similar effect of reductions in FII and FX but not of FVII or FIX on automated thrombin generation and ROTEM clot formation [3, 41]. Based on those experiments, at the activity levels of VKD factors present during maintenance phase warfarin anticoagulation [41] only FII (15–40 u/dL) and FX (11–19 u/dL) would be expected to significantly reduce thrombin generation in vitro (Fig. 1B).

### Is the PT-INR partly to blame for PT-warfarin anticoagulation instability?

Based on the discussion in the previous sections the following issue emerges: The PT-INR that has been the basis of all therapeutic VKA use for seven decades is highly sensitive to reductions of a VKD coagulation factor (FVII) that has little role in bringing about the required antithrombotic effect. Furthermore, due to FVII’s much shorter half-life than that of VKD factors II and X, it is a major cause of short-term variability in the measured PT-INR effect, even within a day. If only FII and X reductions matter, monitoring FVII has little meaningful role but confounds assessment as it exaggerates food and drug interactions and day to day variations in the drug effect.

### The Fiix prothrombin time and Fiix normalized ratio (Fiix-NR)

#### Experimental basis

Our group (PTO, BRG) hypothesized that by ignoring FVII in addition to FIX and measuring only the influence of FII and FX reductions during warfarin monitoring, anticoagulation variability could be decreased, potentially to a degree favorably affecting the efficacy and/or safety of VKA management. In turn a new modified PT was designed that is only sensitive to reductions in FII or FX, called Fiix-PT (pronounced “fix PT”) [3]. The new Fiix-test is not affected by reduced fibrinogen, FV or FVII but only by reductions in factors FII or FX as FIIX-deficient plasma is mixed into diluted test plasma, thereby normalizing all factor levels except those of FII and FX [3, 41]. Other coagulation activators can also be used but when thromboplastin is used to initiate clotting, the Fiix test principle can also be achieved by adding FII, FIX and FX deficient plasma to the test sample and bioequivalent results will be obtained, namely measuring only the influence of FII and FX. A Fiix normalized ratio (Fiix-NR) can then be calculated based on the Fiix-PT ratio in a manner identical to the traditional PT based international normalized ratio (INR, PT-INR) using standards traceable to the WHO international sensitivity index (ISI) standards [7, 44].

**Fig. 2:**
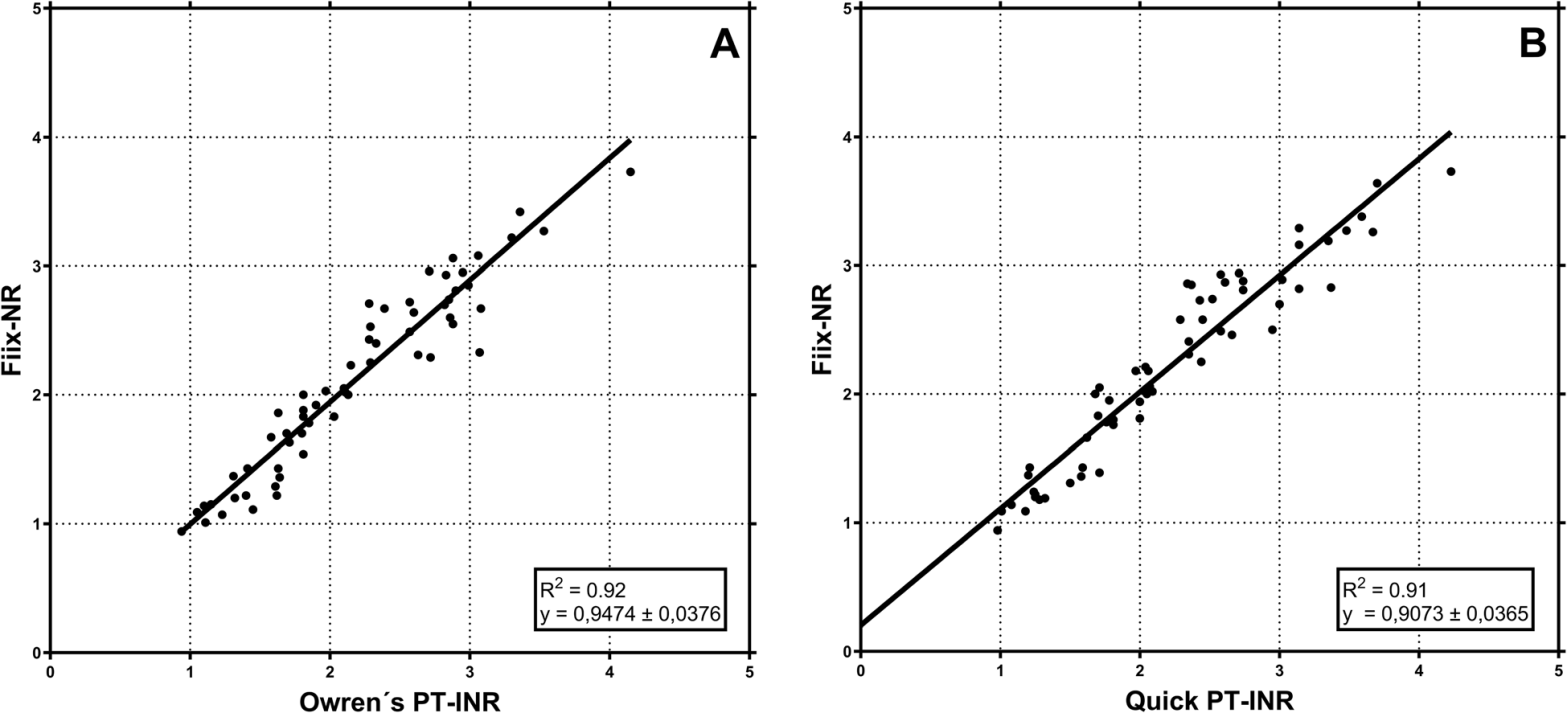
Correlation of Fiix-normalized ratio with undiluted Owren’s PT-INR (SPA, Stago, Asnieres, France: upper panel) or undiluted Quick PT-INR (Neoplastin, Stago, Asnieres, France: lower panel) in samples from patients on stable warfarin therapy. Previously unpublished data from the study described in Thrombosis Research 2012;130:674–81 [3].

**Fig. 3:**
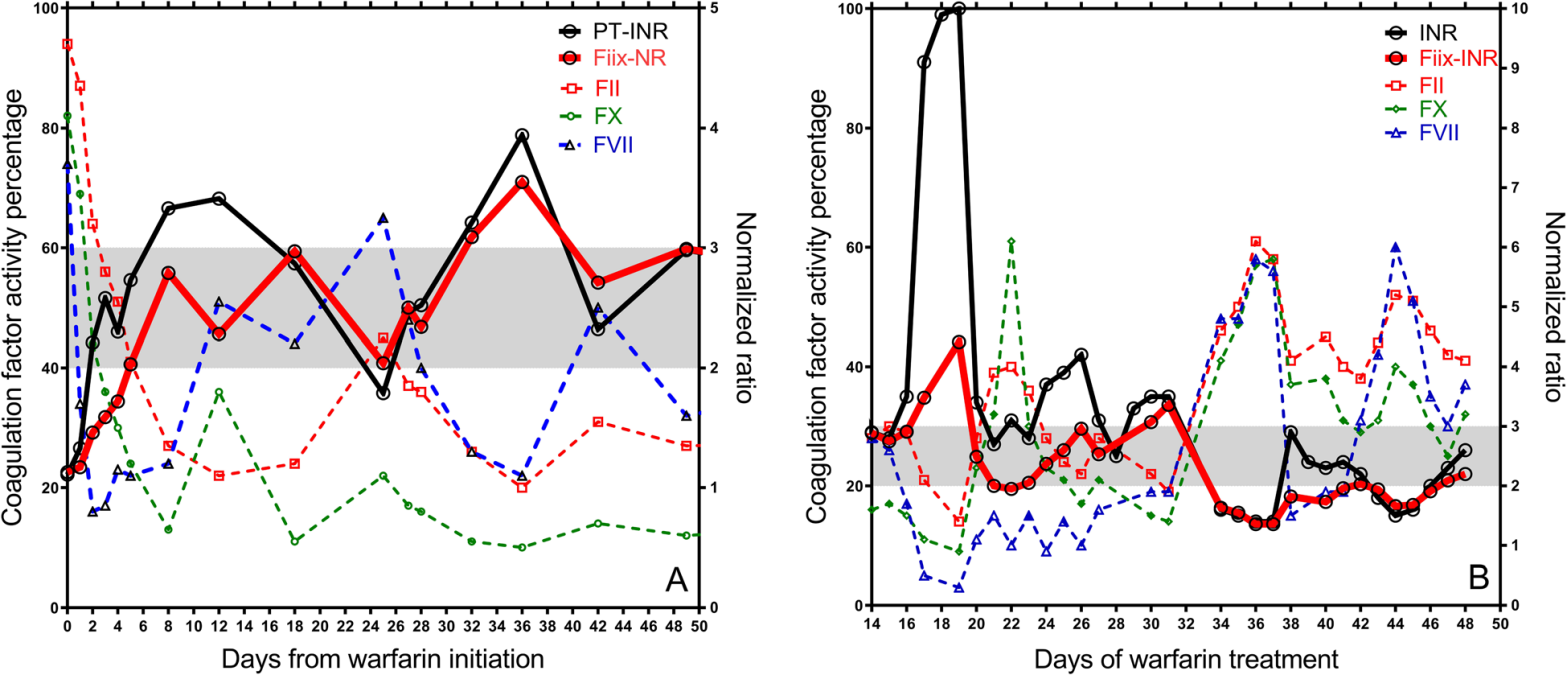
Examples of differences in PT-INR and Fiix-NR during warfarin initiation when factor VII is fluctuating due to dose changes. Both the PT-INR and Fiix-NR were measured using undiluted Neoplastin CI Plus with ISI 1.3 (Diagnostica Stago, Asnieres, France) but in the Fiix-NR measurement factor II and X deficient plasma is added to diluted test plasma prior to addition of Neoplastin. Reproduced with permission from Thrombosis Research 2012;130:674–81 [3]

The Quick-PT and the Owren’s PT have previously been shown to correlate excellently with each other [4]. In samples drawn during stable warfarin management, the Fiix-NR has been shown to correlate well with both the Quick-PT based INR (R^2^ 0.91x; y = 0,91 + 0,20; 49 samples) and the Owren’s PT based INR (R^2^ = 0.92; y = 0.95x + 0.05; 60 samples These previously unpublished data from the study of Gudmundsdottir BR et al. 2012 [3] are shown in Fig. 2 A and B. On the other hand, during warfarin initiation and when factor VII is low for any reason the PT-INR and the Fiix-NR may diverge as illustrated in Fig. 3 [3]..

### Fiixing warfarin management

#### The Fiix trial

**Fig. 4:**
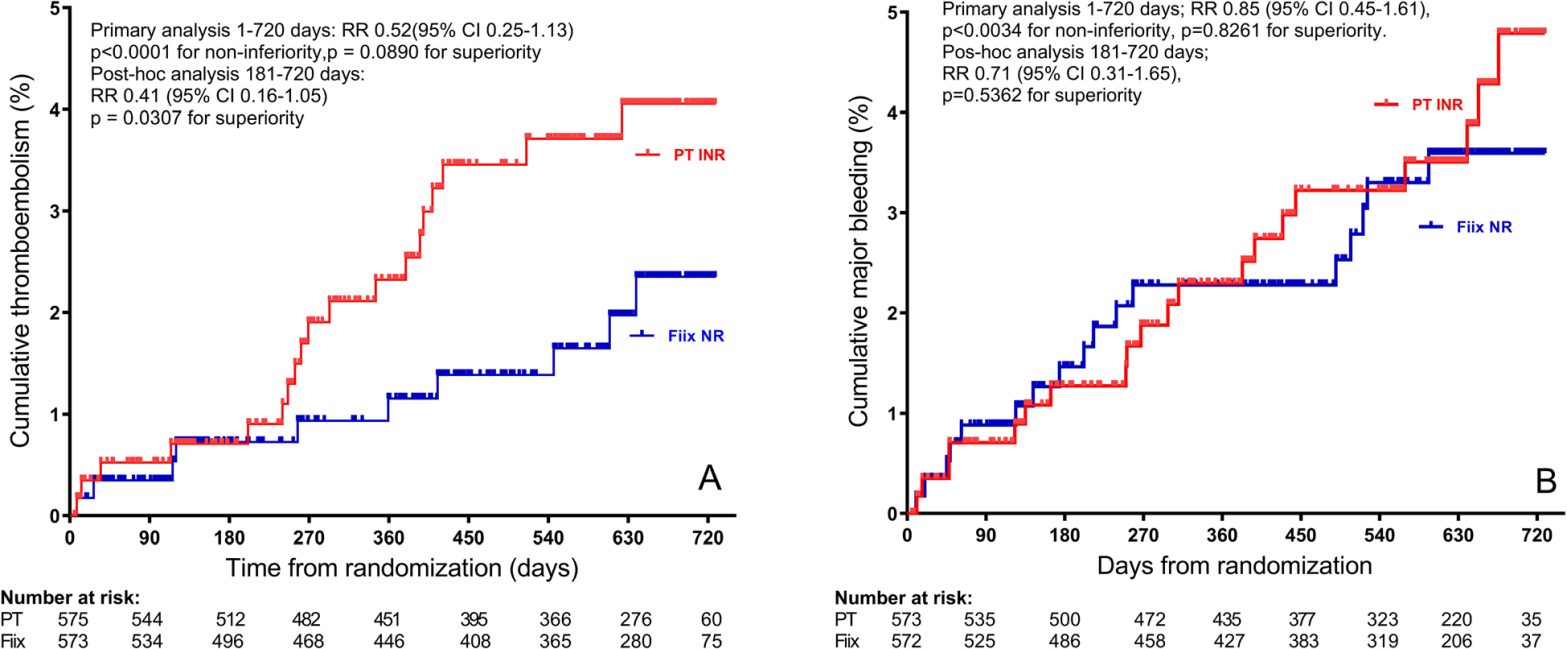
Cumulative non-fatal and fatal thromboembolic events (panel A) and major bleeding (panel B) during treatment days 1–720 of the randomized double blind Fiix trial in patients monitored either with Fiix-warfarin (blue) or PT-warfarin (red). Reproduced with permission from The Lancet Haematology 2015;2:e231-e40 [35].

To test the hypothesis, in an investigator initiated single-center double-blind randomized non-inferiority clinical trial, named the Fiix-trial [35] mostly warfarin-experienced patients with typical mixed indications for anticoagulation were randomized to either Fiix-NR monitoring (Neoplastin and Fiix-deficient plasma, *n* = 573) or standard Quick PT-INR monitoring (Neoplastin, *n* = 575). Fiix-NR or PT-INR (depending on blinded assignment) measured in the central laboratory was reported as a blinded “research INR” to patients, dosing staff and event adjudicators. After a median follow-up of 1.7 years, Fiix-NR anticoagulation variability (variance growth rate) [34], was reduced, TTR was higher (84 vs 80%), and testing and dose adjustments were reduced. Furthermore, a 48% statistically non-inferior reduction was observed in thromboembolic events in Fiix-warfarin patients compared to the PT-warfarin controls (Fig. 4A). As reduced TE became evident only 180 days after the laboratory switched to Fiix-NR monitoring, a post-hoc analysis was performed after excluding the first 180 days and then a statistically superior 59% reduction in TE was observed (P_superiority_ = 0.0307). Despite improved efficacy, major bleeding (MB) was not increased (2.3% per person year in both study arms). With Fiix monitoring, dose change frequency and INR variability were reduced and TTR increased as well, all statistically significantly so. Overall, the Fiix-trial confirmed the hypothesis that the PT-INR is a source of warfarin anticoagulation variability. Further analysis confirmed that higher variability associated with adverse outcomes [37]. One interpretation of these results is that Fiix-warfarin patients have a more consistent anticoagulation level than PT-warfarin patients leading to reduced TE but that reduced variability at the same time may prevent a simultaneous increased bleeding risk.

#### Meta-analysis comparing Fiix-warfarin to PT-warfarin and DOACs in non-valvular atrial fibrillation

Using meta-analytic methods [45], outcomes of Fiix-trial patients with non-valvular AF monitored with PT-warfarin (*n* = 427) or Fiix-warfarin (*n* = 406) were compared to the outcomes of patients with non-valvular AF on PT-warfarin (*n* = 29,272) or on rivaroxaban, apixaban, edoxaban, or dabigatran (n = 42,411) in the pivotal pharmaceutical company initiated RCTs [46–49]. The reported adverse events´ incidence of PT-warfarin control groups in all included trials was similar. This analysis found a statistically significant 49% reduction in composite stroke, systemic embolism or myocardial infarction (MI) in Fiix-warfarin patients when compared to outcome with PT-warfarin control patients in these trials. MB was numerically 37% lower but this finding was non-significant. Point estimates suggested that Fiix-warfarin might also compare favorably to the pooled clinical outcome with the combined DOAC drugs with a 39% reduction in composite stroke or systemic embolism (SSE) or MI and 24% fewer major bleeds but due to limited power these findings were non-significant.

#### Fiix in real world practice

**Fig. 5:**
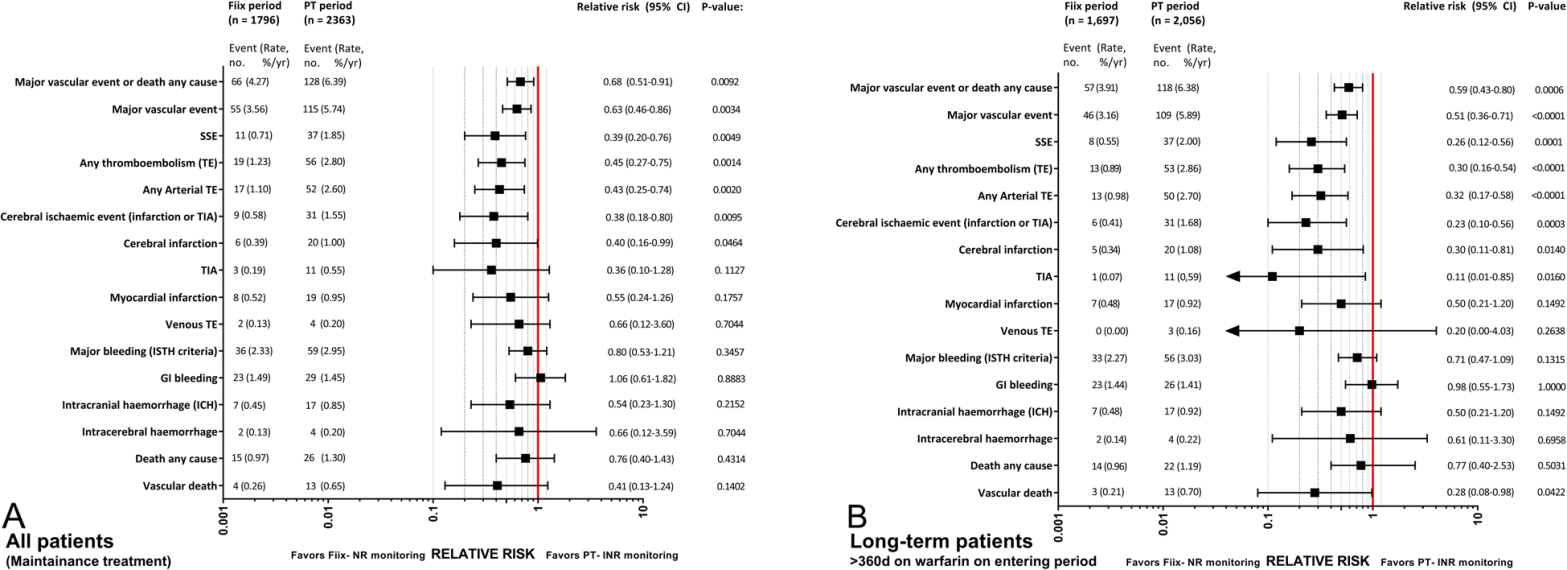
Relative risk plots showing major vascular event rates and relative risk plots comparing clinical outcome during Fiix-NR or traditional PT-INR. Reproduced with permission from Blood 2021;137 [20]:2745–55 [36].

After completing the Fiix-trial in February 2014, all warfarin patients at the Reykjavik center were switched back to the prior Owren’s type PT-INR monitoring. All were then transitioned to Fiix-NR monitoring beginning on July 1st 2016. The effect of the transition was then retrospectively assessed [36]. This real-world single-cohort interrupted time series study assessed the incidence of TE and MB in 2667 patients and compared Fiix-NR to Owren’s PT-INR. Incidence was assessed at monthly intervals during 12 months prior to and 18 months after laboratory switching to Fiix-NR monitoring. Using two-segmented regression, a breakpoint in total TE monthly incidence was seen six months after the change, followed by a 56% reduction in total TE incidence (from 2.82 to 1.23% per patient year, *P* = 0.019; number needed to treat to prevent one TE = 63). After excluding this 6-month transition period, there was no difference in MB between the 12-month Fiix-period (2.3%) and the 12-month PT-period (2.7%, *p* = 0.25). A separate analysis showed that Fiix-monitoring significantly reduced testing, dose adjustments and normalized ratio variability by about one third and increased TTR as well albeit to a minor degree (*P* = 0.0157). A further analysis shown in Fig. 5 demonstrated that the relative risk of suffering any TE, was reduced to 0.45 (0.27–0.75) during Fiix-NR compared to PT-INR monitoring and even more in patients treated long-term. The relative risk of suffering major bleeding was not significantly reduced in that analysis (0.80 (0.53–1.21).

The results of this pre-post study were similar to the Fiix-trial results. Together the two clinical studies suggest that Fiix-NR monitoring is an improvement over monitoring with two standard but different thromboplastin reagents, namely Neoplastin (Quick PT-INR, rabbit brain, ISI 1.3) and the SPA reagent (Owren’s PT-INR, rabbit brain, ISI 1.1).

#### Fiix-warfarin initiation dose protocol

During warfarin initiation, Fiix-NR changes will be observed later than most physicians might predict based on their experience with managing FVII sensitive PT-INRs. This can lead to inaccurate warfarin dose adjustments and early over-anticoagulation. Therefore, a modified initiation algorithm must be used (Table 1) that prevents early over-anticoagulation during Fiix-warfarin initiation [44]. However, during maintenance Fiix-warfarin treatment, the dosing algorithm designed for PT-warfarin patients is appropriate as this did associate with favorable outcomes in our studies [35, 36].

**Table 1:** Old PT-INR based initiation protocol and new initiation protocol modified for the slower responding Fiix prothrombin time (Fiix-NR). Modified from Journal of Thrombosis and Thrombolysis 2019;48:685–9 [44].

Starting daily dose on day 1	Normalized ratio on day 4	Warfarin dose; Old PT-INR initiation protocol (mg/day)	Warfarin dose; New adapted Fiix-NR initiation protocol (mg/day)*
**< 65 year old;** **4 mg daily**			
	< 1.3	6	5
	1.3	4	4
	1.4–1.5	4	3
	1.6–1.7	4	2
	1.8–2.5	2	1.0–1.3
	> 2.5	1.3	Dose skipped and adapted**
**≥65 year old;** **6 mg daily**			
	< 1.3	9	8
	1.3	6	6
	1.4–1.5	6	4.5
	1.6–1.7	6	3
	1.8–2.5	3	1.5–2.0
	> 2.5	2	Dose skipped and adapted**

*The Fiix normalized ratio (Fiix-NR) responds slower than the PT-INR due to insensitivity to factor VII reductions. ** Dose usually skipped for 1–2 days and dose then reduced based on rate of rise of the normalized ratio

#### Other possible uses of the Fiix test: the dilute Fiix-PT (dFiix-PT)

Measuring DOAC anticoagulant effect may have useful applications [50] but no single test can be used for this purpose [50]. A test using highly diluted thromboplastin, the tissue thromboplastin inhibition test (a dilute PT (dPT) assay) used in the past for lupus anticoagulant detection was known to be sensitive to heparin, thrombin and anti-Xa inhibitors but not to pentasaccharide [51]. The sensitivity of both dPT and dilute Fiix-PT clotting times for the detection of different anticoagulants were tested using high dilutions of thromboplastin hypothesizing that the dFiix-PT might be less influenced by confounders. In short, the dFiix-PT at a single thromboplastin dilution could determine warfarin normalized ratios and quantitative concentrations of dabigatran, rivaroxaban, apixaban, unfractionated heparin and enoxaparin but not of fondaparinux. The PT was less effective as two dilutions had to be used and the dPT ratio did not correlate well with the INR in warfarin patient samples [52].

#### Adoption of Fiix-PT into clinical practice

The Fiix test is already available as a CE marked product from a single manufacturer in Europe and can be easily automated. The additional Fiix-reagent (Fiix deficient plasma) adds somewhat to the cost of the reagent compared to traditional PT tests and this may initially be seen as an issue by laboratory directors. However, it must be considered that the benefit of Fiix-NR monitoring is to the patient, health insurance and society due to fewer serious events that have long-term consequences. A cost-benefit study will therefore be important along the way of introducing the Fiix concept to different stakeholders in health care.

## Conclusion

The new Fiix test was based on the hypothesis that monitoring VKAs should focus on the two factors responsible for the antithrombotic effect, namely FII and FX, and that ignoring FVII (in addition to already traditionally ignoring FIX) is reasonable as VKA therapy rarely produces reduction in FVII and FIX activity sufficiently low to be associated with spontaneous bleeding. Two clinical studies now suggest that the hypothesis has merits but further study by independent investigators would be welcomed. As the Fiix-NR is insensitive to factor VII, the measured effect is more stable than was previously achievable and this leads to fewer dose-adjustments and, therefore, less variable warfarin anticoagulation. The TTR was improved as well in the high TTR populations studied, albeit less notable than the variability reduction. In future studies it will be important to assess how Fiix-monitored warfarin compares to clinical outcomes with DOAC drugs. Future studies could also investigate if a lower Fiix-NR target range could improve safety without much loss of efficacy. However, although these findings remain to be externally validated, Fiix-NR monitored warfarin appears to be an improved potentially practice-changing anticoagulant-monitoring test combination compared traditional PT-INR monitored warfarin.

## Nomenclature

Fiix = factors II and X only (pronounced “fix”).

Fiix prothrombin time (Fiix-PT).

Fiix-NR = Fiix normalized ratio.

PT-warfarin = warfarin monitored with traditional PT-INR.

Fiix-warfarin = warfarin monitored with the new Fiix-NR.

VKA = vitamin K antagonists.

VKD = vitamin K dependent.

TTR = time within target INR range by Rosendaal method.

## Data Availability

N.A.

## References

[1] Quick AJ, Stanley-Brown M, Bancroft FW (1935). A study of the coagulation defect in hemophilia and in jaundice. Am J Med Sci.

[2] Owren PA, Aas K (1951). The control of dicumarol therapy and the quantitative determination of prothrombin and proconvertin. Scand J Clin Lab Invest.

[3] Gudmundsdottir BR, Francis CW, Bjornsdottir AM, Nellbring M, Onundarson PT (2012). Critical role of factors II and X during coumarin anticoagulation and their combined measurement with a new Fiix-prothrombin time. Thromb Res.

[4] Haraldsson HM, Onundarson PT, Einarsdottir KA, Gudmundsdottir BR, Petursson MK, Palsson K (1997). Performance of prothrombin-proconvertin time as a monitoring test of oral anticoagulation therapy. Am J Clin Pathol.

[5] Ageno W, Gallus AS, Wittkowsky A, Crowther M, Hylek EM, Palareti G (2012). Oral anticoagulant therapy: antithrombotic therapy and prevention of thrombosis, 9th ed: American College of Chest Physicians Evidence-Based Clinical Practice Guidelines. Chest..

[6] Ansell J, Hirsh J, Hylek E, Jacobson A, Crowther M, Palareti G (2008). Pharmacology and management of the vitamin K antagonists: American College of Chest Physicians Evidence-Based Clinical Practice Guidelines (8th edition). Chest..

[7] Kirkwood TB (1983). Calibration of reference thromboplastins and standardisation of the prothrombin time ratio. Thromb Haemost.

[8] Xi M, Beguin S, Hemker HC (1989). The relative importance of the factors II, VII, IX and X for the prothrombinase activity in plasma of orally anticoagulated patients. Thromb Haemost.

[9] Zivelin A, Rao LV, Rapaport SI (1993). Mechanism of the anticoagulant effect of warfarin as evaluated in rabbits by selective depression of individual procoagulant vitamin K-dependent clotting factors. J Clin Invest.

[10] January CT, Wann LS, Alpert JS, Calkins H, Cigarroa JE, Cleveland JC (2014). 2014 AHA/ACC/HRS guideline for the management of patients with atrial fibrillation: a report of the American College of Cardiology/American Heart Association task force on practice guidelines and the Heart Rhythm Society. J Am Coll Cardiol.

[11] Kearon C, Akl EA, Ornelas J, Blaivas A, Jimenez D, Bounameaux H, Huisman M, King CS, Morris TA, Sood N, Stevens SM, Vintch JRE, Wells P, Woller SC, Moores L (2016). Antithrombotic therapy for VTE disease: CHEST guideline and expert panel report. Chest..

[12] Ruff CT, Giugliano RP, Braunwald E, Hoffman EB, Deenadayalu N, Ezekowitz MD, Camm AJ, Weitz JI, Lewis BS, Parkhomenko A, Yamashita T, Antman EM (2014). Comparison of the efficacy and safety of new oral anticoagulants with warfarin in patients with atrial fibrillation: a meta-analysis of randomised trials. Lancet..

[13] van der Hulle T, den Exter PL, Kooiman J, van der Hoeven JJ, Huisman MV, Klok FA (2014). Meta-analysis of the efficacy and safety of new oral anticoagulants in patients with cancer-associated acute venous thromboembolism. J Thromb Haemost.

[14] Eikelboom JW, Connolly SJ, Brueckmann M, Granger CB, Kappetein AP, Mack MJ, Blatchford J, Devenny K, Friedman J, Guiver K, Harper R, Khder Y, Lobmeyer MT, Maas H, Voigt JU, Simoons ML, van de Werf F (2013). Dabigatran versus warfarin in patients with mechanical heart valves. N Engl J Med.

[15] Pengo V, Denas G, Zoppellaro G, Jose SP, Hoxha A, Ruffatti A, Andreoli L, Tincani A, Cenci C, Prisco D, Fierro T, Gresele P, Cafolla A, de Micheli V, Ghirarduzzi A, Tosetto A, Falanga A, Martinelli I, Testa S, Barcellona D, Gerosa M, Banzato A (2018). Rivaroxaban vs warfarin in high-risk patients with antiphospholipid syndrome. Blood..

[16] Ordi-Ros J, Saez-Comet L, Perez-Conesa M, Vidal X, Riera-Mestre A, Castro-Salomo A (2019). Rivaroxaban versus vitamin K antagonist in Antiphospholipid syndrome: a randomized noninferiority trial. Ann Intern Med.

[17] Pengo V, Hoxha A, Andreoli L, Tincani A, Silvestri E, Prisco D, Fierro T, Gresele P, Cafolla A, de Micheli V, Ghirarduzzi A, Tosetto A, Falanga A, Martinelli I, Testa S, Barcellona D, Gerosa M, Denas G (2021). Trial of rivaroxaban in AntiPhospholipid syndrome (TRAPS): two-year outcomes after the study closure. J Thromb Haemost.

[18] Robinson AA, Trankle CR, Eubanks G, Schumann C, Thompson P, Wallace RL, et al. Off-label Use of Direct Oral Anticoagulants Compared With Warfarin for Left Ventricular Thrombi. JAMA Cardiol. 2020. 10.1001/jamacardio.2020.0652. 10.1001/jamacardio.2020.0652 PMC7177639 32320043

[19] Rosendaal FR, Cannegieter SC, van der Meer FJ, Briet E (1993). A method to determine the optimal intensity of oral anticoagulant therapy. Thromb Haemost.

[20] Wan Y, Heneghan C, Perera R, Roberts N, Hollowell J, Glasziou P, Bankhead C, Xu Y (2008). Anticoagulation control and prediction of adverse events in patients with atrial fibrillation: a systematic review. Circulation Cardiovascular quality and outcomes.

[21] Björck F, Renlund H, Lip GYH, Wester P, Svensson PJ, Själander A (2016). Outcomes in a warfarin-treated population with atrial fibrillation. JAMA Cardiol.

[22] Wallentin L, Yusuf S, Ezekowitz MD, Alings M, Flather M, Franzosi MG, Pais P, Dans A, Eikelboom J, Oldgren J, Pogue J, Reilly PA, Yang S, Connolly SJ (2010). Efficacy and safety of dabigatran compared with warfarin at different levels of international normalised ratio control for stroke prevention in atrial fibrillation: an analysis of the RE-LY trial. Lancet.

[23] Wallentin L, Lopes RD, Hanna M, Thomas L, Hellkamp A, Nepal S, Hylek EM, al-Khatib SM, Alexander JH, Alings M, Amerena J, Ansell J, Aylward P, Bartunek J, Commerford P, de Caterina R, Erol C, Harjola VP, Held C, Horowitz JD, Huber K, Husted S, Keltai M, Lanas F, Lisheng L, McMurray J, Oh BH, Rosenqvist M, Ruzyllo W, Steg PG, Vinereanu D, Xavier D, Granger CB, Apixaban for Reduction in Stroke and Other Thromboembolic Events in Atrial Fibrillation (ARISTOTLE) Investigators (2013). Efficacy and safety of apixaban compared with warfarin at different levels of predicted international normalized ratio control for stroke prevention in atrial fibrillation. Circulation.

[24] Garmendia CA, Nassar Gorra L, Rodriguez AL, Trepka MJ, Veledar E, Madhivanan P (2019). Evaluation of the inclusion of studies identified by the FDA as having falsified data in the results of Meta-analyses: the example of the Apixaban trials. JAMA Intern Med.

[25] Shpak M, Ramakrishnan A, Nadasdy Z, Cowperthwaite M, Fanale C (2018). Higher incidence of ischemic stroke in patients taking novel Oral anticoagulants. Stroke..

[26] Vinogradova Y, Coupland C, Hill T, Hippisley-Cox J (2018). Risks and benefits of direct oral anticoagulants versus warfarin in a real world setting: cohort study in primary care. Bmj..

[27] Holbrook A, Schulman S, Witt DM, Vandvik PO, Fish J, Kovacs MJ, Svensson PJ, Veenstra DL, Crowther M, Guyatt GH (2012). Evidence-based management of anticoagulant therapy: antithrombotic therapy and prevention of thrombosis, 9th ed: American College of Chest Physicians Evidence-Based Clinical Practice Guidelines. Chest.

[28] Owren PA (1947). Parahaemophilia; haemorrhagic diathesis due to absence of a previously unknown clotting factor. Lancet.

[29] Owen CA, Bollman JL (1948). Prothrombin conversion factor of dicumarol plasma. Proceedings of the Society for Experimental Biology and Medicine Society for Experimental Biology and Medicine.

[30] Biggs R, Douglas AS, Macfarlane RG, Dacie JV, Pitney WR (1952). Merskey. Christmas disease: a condition previously mistaken for haemophilia. Br Med J.

[31] Duckert F, Fluckiger P, Matter M, Koller F (1955). Clotting factor X; physiologic and physico-chemical properties. Proceedings of the Society for Experimental Biology and Medicine Society for Experimental Biology and Medicine..

[32] Burn J, Pirmohamed M (2018). Direct oral anticoagulants versus warfarin: is new always better than the old?. Open heart.

[33] Kearon C, Ginsberg JS, Kovacs MJ, Anderson DR, Wells P, Julian JA, MacKinnon B, Weitz JI, Crowther MA, Dolan S, Turpie AG, Geerts W, Solymoss S, van Nguyen P, Demers C, Kahn SR, Kassis J, Rodger M, Hambleton J, Gent M (2003). Comparison of low-intensity warfarin therapy with conventional-intensity warfarin therapy for long-term prevention of recurrent venous thromboembolism. N Engl J Med.

[34] Ibrahim S, Jespersen J, Poller L (2013). European, action, on, et al. the clinical evaluation of international normalized ratio variability and control in conventional oral anticoagulant administration by use of the variance growth rate. J Thromb Haemost.

[35] Onundarson PT, Francis CW, Indridason OS, Arnar DO, Bjornsson ES, Magnusson MK, Juliusson SJ, Jensdottir HM, Vidarsson B, Gunnarsson PS, Lund SH, Gudmundsdottir BR (2015). Fiix-prothrombin time versus standard prothrombin time for monitoring of warfarin anticoagulation: a single Centre, double-blind, randomised, non-inferiority trial. The Lancet Haematology.

[36] Oskarsdottir AR, Gudmundsdottir BR, Jensdottir HM, Flygenring B, Palsson R, Onundarson PT (2021). Ignoring instead of chasing after coagulation factor VII during warfarin management: an interrupted time series study. Blood..

[37] Oskarsdottir AR, Gudmundsdottir BR, Indridason OS, Lund SH, Arnar DO, Bjornsson ES (2017). Reduced anticoagulation variability in patients on warfarin monitored with Fiix-prothrombin time associates with reduced thromboembolism: the Fiix-trial. J Thromb Thrombolysis.

[38] Pirmohamed M. Warfarin: The End or the End of One Size Fits All Therapy? J Pers Med. 2018;8(3). 10.3390/jpm8030022 PMC6163581 29958440

[39] Furie B (2013). Do pharmacogenetics have a role in the dosing of vitamin K antagonists?. N Engl J Med.

[40] Witt DM, Delate T, Clark NP, Martell C, Tran T, Crowther MA (2010). Twelve-month outcomes and predictors of very stable INR control in prevalent warfarin users. J Thromb Haemost.

[41] Jonsson PI, Letertre L, Juliusson SJ, Gudmundsdottir BR, Francis CW, Onundarson PT (2017). During warfarin induction, the Fiix-prothrombin time reflects the anticoagulation level better than the standard prothrombin time. J Thromb Haemost.

[42] Furie B, Diuguid CF, Jacobs M, Diuguid DL, Furie BC (1990). Randomized prospective trial comparing the native prothrombin antigen with the prothrombin time for monitoring oral anticoagulant therapy. Blood..

[43] Furie B, Liebman HA, Blanchard RA, Coleman MS, Kruger SF, Furie BC (1984). Comparison of the native prothrombin antigen and the prothrombin time for monitoring oral anticoagulant therapy. Blood..

[44] Onundarson PT, Gudmundsdottir BR (2019). The need for an adapted initiation nomogram during Fiix prothrombin time monitoring of warfarin. J Thromb Thrombolysis.

[45] Onundarson PT, Arnar DO, Lund SH, Gudmundsdottir BR, Francis CW, Indridason OS (2016). Fiix-prothrombin time monitoring improves warfarin anticoagulation outcome in atrial fibrillation: a systematic review of randomized trials comparing Fiix-warfarin or direct oral anticoagulants to standard PT-warfarin. Int J Lab Hematol.

[46] Connolly SJ, Ezekowitz MD, Yusuf S, Eikelboom J, Oldgren J, Parekh A, Pogue J, Reilly PA, Themeles E, Varrone J, Wang S, Alings M, Xavier D, Zhu J, Diaz R, Lewis BS, Darius H, Diener HC, Joyner CD, Wallentin L (2009). Dabigatran versus warfarin in patients with atrial fibrillation. N Engl J Med.

[47] Patel MR, Mahaffey KW, Garg J, Pan G, Singer DE, Hacke W, Breithardt G, Halperin JL, Hankey GJ, Piccini JP, Becker RC, Nessel CC, Paolini JF, Berkowitz SD, Fox KAA, Califf RM, the ROCKET AF Steering Committee (2011). Rivaroxaban versus warfarin in nonvalvular atrial fibrillation. N Engl J Med.

[48] Granger CB, Alexander JH, McMurray JJ, Lopes RD, Hylek EM, Hanna M (2011). Apixaban versus warfarin in patients with atrial fibrillation. N Engl J Med.

[49] Giugliano RP, Ruff CT, Braunwald E, Murphy SA, Wiviott SD, Halperin JL, Waldo AL, Ezekowitz MD, Weitz JI, Špinar J, Ruzyllo W, Ruda M, Koretsune Y, Betcher J, Shi M, Grip LT, Patel SP, Patel I, Hanyok JJ, Mercuri M, Antman EM (2013). Edoxaban versus warfarin in patients with atrial fibrillation. N Engl J Med.

[50] Onundarson PT, Flygenring B (2019). Oral anticoagulant monitoring: are we on the right track?. Int J Lab Hematol.

[51] Gerbutavicius R, Iqbal O, Messmore HL, Wehrmacher WH, Hoppensteadt DA, Gerbutaviciene R, Griniute R, Fareed J (2003). Differential effects of DX-9065a, argatroban, and synthetic pentasaccharide on tissue thromboplastin inhibition test and dilute Russell's viper venom test. Clin Appl Thromb Hemost.

[52] Letertre LR, Gudmundsdottir BR, Francis CW, Gosselin RC, Skeppholm M, Malmstrom RE, Moll S, Hawes E, Francart S, Onundarson PT (2016). A single test to assay warfarin, dabigatran, rivaroxaban, apixaban, unfractionated heparin, and enoxaparin in plasma. J Thromb Haemost.

